# SARS-CoV-2 infection prevalence and associated factors: a serial population-based study in Espírito Santo, Brazil, May to June 2020

**DOI:** 10.1590/S1679-49742022000200023

**Published:** 2022-08-29

**Authors:** Orlei Amaral Cardoso, Cristiana Costa Gomes, Crispim Cerutti, Ethel Leonor Noia Maciel, Filomena Euridice Carvalho de Alencar, Gilton Luiz Almada, Laylla Ribeiro Macedo, Letícia Tabachi Silva, Nésio Fernandes de Medeiros, Pablo Medeiros Jabor, Raphael Lubiana Zanotti, Tania Reuter, Vera Lucia Gomes de Andrade, Whisllay Maciel Bastos, Eliana Zandonade

**Affiliations:** 1 Secretaria de Estado da Saúde do Espírito Santo, Subsecretaria de Estado de Vigilância em Saúde, Vitória, ES, Brazil; 2 Universidade Federal do Espírito Santo, Departamento de Medicina Social, Vitória, ES, Brazil; 3 Universidade Federal do Espírito Santo, Laboratório de Epidemiologia, Vitória, ES, Brazil; 4 Universidade Federal do Espírito Santo, Departamento de Pediatria, Vitória, ES, Brazil; 5 Secretaria de Estado da Saúde do Espírito Santo, Centro de Informações Estratégicas de Vigilância em Saúde, Vitória, ES, Brazil; 6 Governo do Estado do Espírito Santo, Instituto Jones dos Santos Neves, Vitória, ES, Brazil; 7 Instituto Jones dos Santos Neves, Coordenação de Geoespacialização, Vitória, ES, Brazil; 8 Universidade Federal do Espírito Santo, Hospital Universitário Cassiano Antônio de Moraes, Vitória, ES, Brazil; 9 Secretaria de Estado da Saúde do Tocantins, Diretoria Geral de Vigilância em Saúde, Palmas, TO, Brazil; 10 Universidade Federal do Espírito Santo, Departamento de Estatística, Vitória, ES, Brazil

**Keywords:** Coronavirus Infections, COVID-19, Cross-Sectional Studies, Epidemiological Surveys

## Abstract

**Objective:**

To analyze SARS-CoV-2 seroprevalence and association of sociodemographic and clinical aspects in the state of Espírito Santo, Brazil.

**Methods:**

This was a serial cross-sectional study carried out in four phases, using households as the unit of analysis, from May to June 2020. Eleven municipalities were surveyed, with a sample of 4,500 households in each phase.

**Results:**

Prevalence ranged from 2.1% (95%CI 1.7;2.5) on May 10 (first phase) to 9.6% (95%CI 8.8;10.4) on June 21 (fourth phase). In the Greater Vitória Metropolitan Region, the prevalence were 2.7% (95%CI 2.2;3.3) in the first phase, and 11.5% (95%CI 10.5;12.6) in the fourth phase; in the interior region of the state, prevalence ranged from 0.4% (95%CI 0.1;0.9) to 4.4% (95%CI 3.2;5.5) between the two phases.

**Conclusion:**

The increase in SARS-CoV-2 seroprevalence found in the fourth phase highlighted the high transmission of the virus, information that can support management of the pandemic.

**Study contributions**

**Main results**

An increase in SARS-CoV-2 infection prevalence was found in Espírito Santo state as a whole, in Greater Vitória and in the interior region of the state. The odds of reactive tests were higher for females and in households with more than two residents.

**Implications for services**

Population-based sero-surveys can indicate safer ways for the adoption of measures, enabling knowledge of the characteristics of the population assessed, both from the clinical and the socioeconomic point of view.

**Perspectives**

Studies that aim to provide knowledge on the spread of the SARS-CoV-2 virus can guide pandemic management processes, reinforcing the importance of conducting more sero-surveys, including genome mapping.

## INTRODUCTION

Following the first cases of COVID-19, an infection caused by SARS-CoV-2, in China in late 2019, COVID-19 cases and deaths were soon reported on all continents,^1^ ^-^ ^4^ given the rapid transmission of this virus via saliva droplets or aerosols.^5^ ^,^ ^6^ With effect from the declaration of the pandemic on March 11, 2020, governments were obliged to encourage changes in behaviors within society and to make decisions in order to mitigate the effects of the pandemic on the population.^6^

The return to social and work activities after a period of strict isolation measures led to an increase in COVID-19 incidence and mortality curves, after an initial period of decline in these indicators worldwide. This oscillation in the epidemiological profile of the disease creates uncertainty about the availability of resources necessary for the adequate care of cases.^7^

Data released by the Brazilian Ministry of Health showed that by September 12, 2021, approximately 20 million confirmed COVID-19 cases and approximately 600,000 COVID-19 deaths had been reported in Brazil as a whole; in the state of Espírito Santo, as at the same date, 571,396 COVID-19 cases and 12,352 COVID-19 deaths had been reported.^8^

In this context of the COVID-19 pandemic, information about its incidence rate and the population’s immunity status is important for supporting the planning of public policies aimed at controlling the disease. Population-based surveys are useful for monitoring the progression of infection, gaining knowledge on and/or monitoring characteristics/behaviors of the population and/or health services, in the face of the spread of the virus. In this sense, the carrying out of population-based prevalence studies has been indicated by the World Health Organization (WHO) because they assist in health authority decision-making, especially when performed serially, enabling assessment of the behavior of the disease over time.^9^ ^,^ ^10^

This study aimed to analyze SARS-CoV-2 seroprevalence and its association with sociodemographic and clinical aspects, in the state of Espírito Santo, Brazil.

## METHODS

This was a population-based, serial, cross-sectional study conducted in Espírito Santo state, taking households as the unit of analysis. It was designed according to the guidelines established in the population-based age-stratified seroepidemiological investigation protocol for COVID-19 virus infection.^9^

Four sequential cross-sectional surveys were conducted, referred to here as ‘phases’. The sampling process for each phase was independent. The interval between phases was 15 days, and all four were completed within two months. The phases began on May 10, May 24, June 7, and June 21, 2020, with one week of data collection for each phase.

According to the Brazilian Institute of Geography and Statistics (IBGE), Espírito Santo had 4,018,650 inhabitants in 2019, living in four intermediate regions and eight immediate regions.^11^ The eight strata of the survey correspond to the state’s immediate regions: Vitória, comprising 10 municipalities; Afonso Cláudio-Venda Nova do Imigrante-Santa Maria de Jetibá, with 11; São Mateus, with 9; Linhares, with 6; Colatina, with 13; Nova Venécia, with 5; Cachoeiro de Itapemirim, with 12; and Alegre, also comprising 12 municipalities.^11^

Sampling was carried out in sentinel municipalities that concentrate the largest urban populations per geographic region of the state. The selection of sentinel municipalities was justified by the short period of time and limited availability of tests. The most representative municipalities of the immediate regions were selected (one for each region), to which the most populous municipalities of the Greater Vitória Metropolitan Region were added (Vitória; Vila Velha; Cariacica; Serra). In this way, 11 municipalities were surveyed, and the results are presented according to three clusters: the whole of Espírito Santo state; the Greater Vitória Metropolitan Region; and municipalities in the interior region of the state. 

The sample size for each phase was set at 4,500 households, taking expected prevalence for each phase to be 3%, 5%, 10%, and 20%, respectively. Total precision associated with these sample sizes was 0.5, 0.6, 1.0, and 1.2 percentage points, respectively. A 5% significance level was adopted. 

The number of households selected in each municipality was proportional to the size of its urban population. Census tracts were used as territorial units, taking urban census tracts according to the census tract grid established in 2010, whereby the inclusion criteria were tract size of less than 100 hectares and more than 200 households in the tract. When comparing the 2010 census tract grid with the preliminary grid for 2020, minor changes were found in the census tract division. A fixed number of households in the sample of 40 per census tract was adopted, resulting in a final sample of more than 4,500 individuals, despite rounding. A larger number of census tracts was selected in municipalities with larger populations, in order to ensure sample proportionality. As recommended by the IBGE, we used census tracts in order to obtain population homogeneity.

The households were selected systematically, by selecting one household in five, starting from a randomly generated point. In each household selected for the sample, a list of residents was made and only one of them was randomly selected to participate in the survey, in order to ensure the independence of the sampling units considered in the study, i.e. the households. At each new phase of the survey, sampling included the same census tracts, but different households to those included in the previous phases. If no one was living in a selected household at the time of the survey, the researcher moved on to the next household and then continued the systematic selection approach. If this resulted in selection of a household already selected in a previous phase, the researcher moved on to the next household. Individuals above 2 years of age were included in the study. 

The data were collected by means of interviews. In the case of respondents under 16 years old, the questions were answered by their legal guardians.

The following individual data on each participant was obtained in the interviews:

- sex (male; female);
- age group (in years: up to and including 20; 21-40; 41-60; 61-80; 81 and over);
- years of study of the respondent (illiterate; up to 8; 9 or more);
- schooling of the person with the highest level of education in the household (illiterate; elementary education; high school education; complete higher education; incomplete higher education);
- self-reported race/skin color (White; mixed race; Black; Asian; Indigenous);
- number of residents in the household (1; 2; 3; 4; 5 or more);
- going to a health center because of COVID-19 symptoms in the last 15 days (yes; no); and
- COVID-19 symptoms (cough, fever, tiredness, pains, breathing difficulty, changes in taste or smell, other) in the 15 days prior to the interview.

Blood samples were collected by digital puncture with a sterile lancet, according to the technique recommended by the pharmaceutical company, respecting biosafety precautions. IgM and IgG anti-SARS-Cov-2 antibodies were tested for using the Celer rapid immunochromatographic test, registered with the National Health Surveillance Agency (Agência Nacional de Vigilância Sanitária - Anvisa) under No. 80537410048. Tests were considered to be reactive when they indicated a reactive result for SARS-CoV-2 antibodies in the sample, regardless of being IgG or IgM. This test has 86.4% sensitivity and 97.6% specificity.^12^

Data collection was performed using the SUS Primary Health Care platform (e-SUS) and smartphones connected to the internet, with the possibility of data management in the absence of a remote connection. These data generated an Excel spreadsheet file, with subsequent analysis using the Statistical Package for the Social Sciences (SPSS) version 20.0.

The raw data were organized in frequency tables, and prevalence, with its respective 95% confidence intervals (95%CI), was estimated according to points. Analysis of association of the participants’ characteristics with the presence of antibodies - anti-SARS-Cov-2 - was performed using Pearson’s chi-square test and odds ratios (OR), through logistic regression. In the multivariable logistic regression analysis, independent variables that had a p-value in the chi-square test less than or equal to 0.20 for their univariate relationship with the outcome (i.e., odds of a reactive COVID-19 test) were kept in the final model. Adjustment of the effect of each independent variable on the odds of the outcome was performed considering all other variables in the model, concomitantly. The Hosmer-Lemeshow test (HL test) was performed to assess how well the data fitted the model. A 5% significance level was adopted.

The study was approved by the Universidade de Vila Velha Research Ethics Committee: Certificate of Submission for Ethical Appraisal No. 31417020.3.0000.5064; Opinion No. 4.317.264, issued on May 4, 2020. All participants were informed about the survey’s objectives, risks and benefits. Data collection was performed after participants, or their legal guardians in the case of those under 18 years old, had read and signed an Informed Consent Form.

Appropriate biosafety measures were taken to safeguard the health of field workers during data and sample collection. Municipal health services were notified of positive cases in order for the necessary measures to be taken. In households where participants had reactive COVID-19 results or symptomatic cases were detected, tests were offered to the remaining residents. These results were not considered in the prevalence calculation presented, since these individuals were not included in the study sample; however we have presented the ratio between the number of reactive contacts divided by the number of reactive cases, among those selected in the sample.

## RESULTS

The total sample was composed of 18,791 individuals, namely: 4,597 in phase 1, 4,638 in phase 2, 4,633 in phase 3, and 4,923 in phase 4. 

We found a total of 1,148 individuals with reactive results. In households where an individual had a positive test result, all residents present at the time of the survey were tested, totaling 1,826 additional tests; of these, 738 had reactive results, indicating 40.4% reactive results among contacts. 

The majority of the participants were female (62.4%), there was a greater proportion of participants between 41 and 60 years old (35.1%), of mixed race/skin color (45.4%), with nine or more years of study (59.5%), with no COVID-19 symptoms (61.2%) and did not seek the services of a health center when they had COVID-19 symptoms (82.6%) (Table 1). 

**Table 1:** - Distribution of sociodemographic characteristics according to SARS-CoV-2 test result (N=18,791), Espírito Santo, May-June/2020

Variable	Total		Test result				p-value^a^
			Reactive		Non-reactive		
	n	%	n	%	n	%	
Sex							
Female	11,715	62.4	761	6.5	10,954	93.5	0.005
Male	7,071	37.6	387	5.5	6,684	94.5	
Age group (in years)							
≤ 20	1,298	6.9	75	5.8	1,223	94.2	0.028
21-40	5,638	30.1	373	6.6	5,265	93.4	
41-60	6,598	35.1	424	6.4	6,174	93.6	
61-80	4,648	24.7	247	5.3	4,401	94.7	
≥ 81	604	3.2	29	4.8	575	95.2	
Race/skin color							
White	7,291	39.1	371	5.1	6,920	94.9	0.001
Mixed race	8,475	45.4	542	6.4	7,933	93.6	
Black	2,650	14.2	208	7.8	2,442	92.2	
Asian	183	1.0	14	7.7	169	92.3	
Indigenous	49	0.3	3	6.1	46	93.9	
Years of study							
Illiterate	662	3.6	33	5.0	629	95.0	0.089
≤ 8	6,840	36.9	451	6.6	6,389	93.4	
≥ 9	11,050	59.5	655	5.9	10,395	94.1	
Number of residents in the household							
1	2,190	11.7	88	4.0	2,102	96.0	0.001
2	5,064	27.0	251	5.0	4,813	95.0	
3	5,012	26.6	299	6.0	4,713	94.0	
4	3,812	20.3	263	6.9	3,549	93.1	
≥ 5	2,706	14.4	247	9.1	2,459	90.9	
Highest level of schooling in the household							
Illiterate	375	2.0	15	4.0	360	96.0	0.001
Elementary education	4,608	24.5	286	6.2	4,322	93.8	
High school education	7,720	41.1	568	7.4	7,152	92.6	
Complete higher education	4,735	25.2	200	4.2	4,535	95.8	
Incomplete higher education	1,348	7.2	79	5.9	1,269	94.1	
Number of symptoms							
None	11,515	61.2	349	3.0	11,166	97.0	0.001
1	2,999	16.0	165	5.5	2,834	94.5	
2	1,573	8.4	125	7.9	1,448	92.1	
3	901	4.8	103	11.4	798	88.6	
4	1,798	9.6	406	22.6	1,392	77.4	
Went to a health center							
No	15,512	82.6	735	4.7	14,777	95.3	0.001
Yes	3,274	17.4	413	12.6	2,861	87.4	

a) Pearson’s chi-square test.

**Figure 1:**
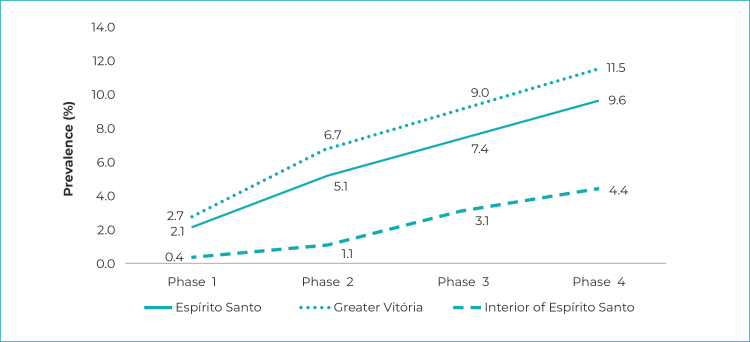
SARS-CoV-2 prevalence, according to data from the Infection Prevalence Survey, Espírito Santo, Greater Vitória and interior region of the state, May-June/2020

Notes: Phase 1 = started on May 10, 2020; Phase 2 = started on May 24, 2020; Phase 3 = started on June 7, 2020; Phase 4 = started on June 21, 2020.

**Figure 2:**
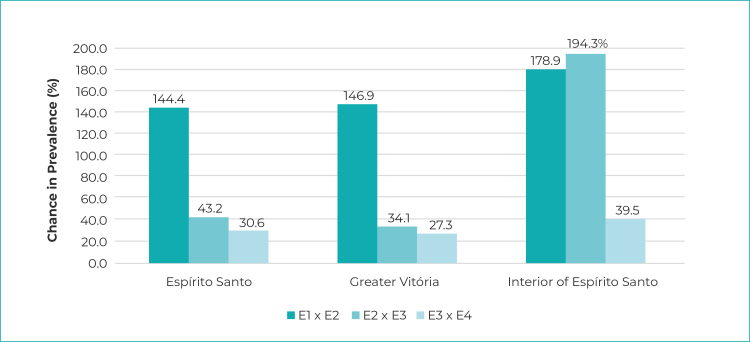
- Percent change in SARS-CoV-2 prevalence, in relation to the previous phase of the Infection Prevalence Survey, Espírito Santo, Greater Vitória and interior region of the state, May-June/2020

Legend: E1 x E2 = Phase 1 in relation to Phase 2; E2 x E3 = Phase 2 in relation to Phase 3; E3 x E4 = Phase 3 in relation to Phase 4 (started on April 21, 2020).

Notes: Phase 1 = started on started on May 10, 2020; Phase 2 = started on May 24, 2020; Phase 3 = started on June 7, 2020; Phase 4 = started on June 21, 2020.

In all three clusters - the state of Espírito Santo, Greater Vitória, interior region of the state -, prevalence rose during the four phases; in the state as a whole, they ranged from 2.1% (95%CI 1.7;2.5) in phase 1 to 9.6% (95%CI 8.8;10.4) in phase 4. Seroprevalence of SARS-Cov-2 infection in the Greater Vitória Metropolitan Region was 2.7% (95%CI 2.2;3.3) in phase 1, reaching 11.5% (95%CI 10.5;12.6) in phase 4. In the interior region of the state, prevalence ranged from 0.4% (95%CI 0.1;0.9) in phase 1 to 4.4% (95%CI 3.2;5.5) in phase 4 (Figure 1). The highest increases occurred between the first and second phases, reaching 179% in the interior region of the state. The increase was less pronounced between second and the third phase, both for Espírito Santo as a whole and for the Greater Vitória Metropolitan Region. The same did not occur in the interior of the state, where an increase of 194% in the prevalence of infection was recorded. Similarly, from the third to the fourth phase, the increases were smaller, ranging from 27% in Greater Vitória to 39% in the interior (Figure 2).

The ratio between the number of reactive contacts (738) and the number of reactive cases in the sample (1,148) was 0.64, indicating less than one reactive contact per reactive case.

Table 2 presents the results of the crude and adjusted odds ratios. The odds of reactive tests increased by 20% for females (OR = 1.20; 95%CI 1.05;1.36), by 18% for individuals with up to 8 years of schooling, by 14% for those with nine years or more of schooling, by 26% for individuals living in households with two residents and by 132% for those living in households with four residents, compared to households with just one resident (p-value = 0.001). The result of the Hosmer-Lemeshow test model fit statistics indicates that the model had goodness of fit (chi-square = 3.864; p-value = 0.869).

**Table 2:** - Sociodemographic factors associated with reactive SARS-CoV-2 test results, according to data from the Infection Prevalence Survey (n=1,148), Espírito Santo, May-June/2020

Variable	Crude model		Adjusted model^b^	
	OR^a^ (95%CI)	p-value	OR^a^ (95%CI)	p-value^c^
Sex				
Male	1.00	0.005	1.00	0.006
Female	1.20 (1.06;1.36)		1.20 (1.05;1.36)	
Age group (in years)				
≤ 20	1.00	0.001	1.00	0.363
21-40	1.16 (0.89;1.49)		1.21 (0.93;1.57)	
41-60	1.12 (0.87;1.44)		1.23 (0.95;1.58)	
61-80	0.92 (0.70;1.19)		1.09 (0.83;1.43)	
≥ 81	0.82 (0.53;1.28)		1.05 (0.67;1.64)	
Race/skin color				
White	0.68 (0.45;1.02)	0.001	0.69 (0.43;1.09)	0.543
Mixed race	0.87 (0.58;1.30)		0.78 (0.49;1.23)	
Black	1.08 (0.71;1.64)		0.95 (0.59;1.52)	
Other	1.00		1.00	
Years of study				
Illiterate	1.00	0.001	1.00	0.024
≤ 8	1.35 (0.94;1.93)		1.18 (0.77;1.82)	
≥ 9	1.20 (0.84;1.72)		1.14 (0.73;1.77)	
Number of residents in the household				
1	1.00	0.001	1.00	0.001
2	1.25 (0.97;1.60)		1.26 (0.98;1.63)	
3	1.52 (1.19;1.93)		1.55 (1.20;2.00)	
4	1.77 (1.38;2.27)		1.79 (1.38;2.32)	
≥ 5	2.40 (1.87;3.08)		2.35 (1.81;3.04)	
Highest level of schooling in the household				
Illiterate	1.00		1.00	
Elementary education	1.59 (0.93;2.70)	0.089	1.18 (0.63;2.20)	0.652
High school education	1.91 (1.13;3.22)		1.27 (0.68;2.40)	
Complete higher education	1.06 (0.62;1.81)		0.75 (0.39;1.44)	
Incomplete higher education	1.49 (0.85;2.63)		1.00 (0.51;1.96)	

a) OR: odds ratio; b) Adjustment performed for all variables included in the model; c) Significance of Pearson`s chi-square test for adjusted ORs.

**Figure 3:**
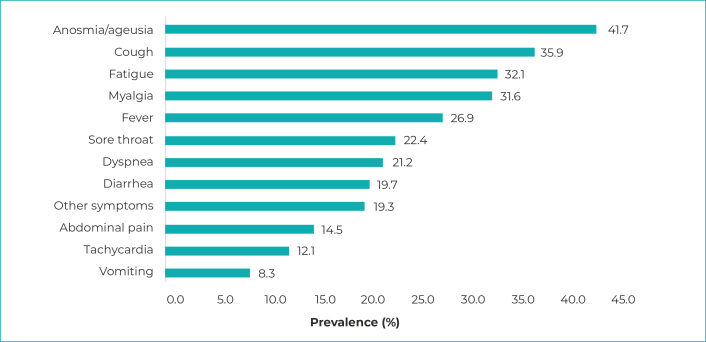
- Most prevalent clinical manifestations in SARS-CoV-2 reactive cases, according to data from the Infection Prevalence Survey, Espírito Santo, May-June/2020

The most prevalent clinical manifestations among reactive cases were anosmia/ageusia (41.7%), cough (35.9%) and fatigue (32.1%). The least prevalent manifestation was vomiting: 8.3% of cases (Figure 3).

## DISCUSSION

Through its four phases, the survey assessed a total of 18,791 individuals, showing an increase in SARS-CoV-2 infection prevalence between each phase and the next, throughout the survey and in all the cluster areas studied: Espírito Santo state as a whole, the Greater Vitória Metropolitan Region, and the interior region of the state. In addition, most of those assessed were female, aged 41 to 60 years, of mixed race/skin color, and with no COVID-19 symptoms. The odds of reactive tests were higher for females, individuals with nine years schooling or more, and those whose household had more than two residents.

This study has some limitations. It is important to note that as interviews were the basis for data collection, there is information bias, both on the part of the interviewers and the interviewees with regard to their memory bias for the latter. There is also selection bias, represented by selective survival: as this was a household-based survey, it could have included disproportionately more individuals from within the mild COVID-19 spectrum, given the greater possibility of hospitalization and/or death among those with the severe clinical form of the disease, which could mean that the prevalence found in this study is underestimated. 

There are concerns about rapid serological tests but these relate to their use in clinical decision making at the individual level, given the need for indication according to the stage of the disease.^12^ ^-^ ^15^ Rapid test administration, for population-based estimates and particularly for monitoring trends over time, was the method chosen by us, because at the time of the survey, there were still no vaccines, antiviral medication, or any specific treatment for COVID-19 available (study phase).^12^ The fact of the test having sensitivity of less than 90% may have made false negative results possible; however, even in these cases, low prevalence probably maintained a high negative predictive value, i.e., high probability of the absence of disease when the test is negative.

It is noteworthy that at the time this study was conducted, in late June 2020, few population-based surveys on SARS-CoV-2 prevalence had been conducted in Brazil, and recommended social distancing was the main measure adopted.^16^ ^,^ ^17^

A study carried out in the state of Rio Grande do Sul showed a prevalence curve that also increased until the third phase, being 0.048% in the first phase, between April 11 and 13, 0.135% in the second phase, from April 25 to 27, and 0.222% in the third phase, from May 9 to 11, 2020.^18^ The prevalence found by that study, in all phases, were lower in relation to those obtained in Espírito Santo, and one of the justifications for these results would be that the data were obtained in a period prior to the epidemic phase in Brazil, as well as the fact that adherence to social distancing measures was greater in Rio Grande do Sul, in relation to what occurred in other parts of Brazil.^12^

Regarding the COVID-19 indicators in Espírito Santo, during the research period, the epidemiological bulletin published on June 24, 2020 indicated that 34,866 cases of the disease had been reported in the state, up to that date, and 1,328 deaths had been recorded, implying a 3.81% case fatality ratio. Standing out among the main measures adopted by the Espírito Santo state health service management at that time are the following: publication of the State Plan for Prevention and Control of the Novel Coronavirus; creation of the Public Health Emergency Operations Center; suspension of teaching activities in public and private education facilities, suspension of events and activities with audiences, besides temporary closure of commercial establishments.^19^

 A study conducted in Teresina, capital city of the state of Piauí, found that over seven weeks with serial testing, between April 19 and May 31, 2020, serological positivity increased from 0.56% (95%CI 0.18;1.30) to 8.33% (95%CI 6.61;10.33).^20^ Moreover, a population-based household survey conducted in the state of Maranhão between July 27, 2020 and August 8, 2020, interviewed 3,156 individuals and found that overall seroprevalence of SARS-CoV-2 antibodies was 40.4% (95%CI 35.6;45.3).^21^

Among the participants who tested positive, there was a predominance of females, five or more residents in the same household and a higher level of education, compared to those who tested negative. At the beginning of the epidemic, cases in Brazil were related to the middle and upper social classes, with a history of returning from countries, mainly European, where the number of COVID-19 cases was high. Over the course of the epidemic, this changed so that the population with lower purchasing power became more affected, which can also be seen in Espírito Santo, reflecting the national scenario.^22^ ^,^ ^23^

Data collected in Chicago, United States, on April 20, 2020, showed a disproportionately higher number of COVID-19 cases among African Americans and the poor: significant spatial clustering of social vulnerability and risk factors were found, both of which were significantly associated with increased COVID-19 mortality rates.^24^ As such, the novel coronavirus pandemic is a challenge for countries where there are profound social inequalities.^25^

In Brazil, it is known that specific groups also suffer the impacts of the pandemic more severely. A study carried out in the country’s five major regions showed that the proportion of individuals with reactive tests was higher among Indigenous, Black and mixed race people, in comparison to White people, besides being inversely associated with socioeconomic status.^26^ ^,^ ^27^

In this study, besides race/skin color disparities among reactive cases, we found a higher percentage of females in the households, reflecting the profile of the caregiver. Whether in family care, household management, or involvement in community initiatives, females are potentially more exposed to the virus, a fact further reinforced by the fact that they are the majority among health workers.^28^ This higher prevalence among females may also be associated with survival bias, since males are at higher risk of progression to the severe form of COVID-19 and/or death due to COVID-19 when compared to females.^29^

We found higher prevalence of SARS-CoV-2 positivity in households with a higher number of residents. The precariousness of housing in some regions, lack of access to basic sanitation, mains water and household sewers, make it difficult to control the epidemic, imposing barriers to ensuring minimum home isolation. The large proportion of households located in substandard settlements has been described by the IBGE on other occasions, and especially in Espírito Santo, this percentage is higher than in most Brazilian states, coming second only to the state of Amazonas.^30^

Finally, it is important to consider that the results of population-based sero-surveys may indicate safer ways for adopting measures that enable knowledge of the characteristics of the population assessed, not only from the clinical point of view but also from the socioeconomic point of view as well. This and future studies that contribute to knowledge about the spread of the virus will be able to provide more precise guidance on COVID-19 pandemic management processes.
